# First Canadian records of genera *Apimela* Mulsant & Rey and *Gyronycha* Casey from New Brunswick: description of two new species and new provincial distribution records (Coleoptera, Staphylinidae, Aleocharinae)

**DOI:** 10.3897/zookeys.672.12488

**Published:** 2017-05-03

**Authors:** Jan Klimaszewski, Reginald P. Webster, Adriano Zanetti, Caroline Bourdon

**Affiliations:** 1 Natural Resources Canada, Canadian Forest Service, Laurentian Forestry Centre, 1055 du P.E.P.S., P.O. Box 10380, Stn. Sainte-Foy, Quebec, Quebec, Canada G1V 4C7; 2 24 Mill Stream Drive, Charters Settlement, NB, Canada E3C 1X1; 3 Museo Civico di Storia Naturale, Lungadige Porta Vittoria 9, I-37129 Verona, Italy

**Keywords:** Coleoptera, Staphylinidae, Aleocharinae, *Apimela*, *Gyronycha*, taxonomy, new species, Canada

## Abstract

Two genera, *Apimela* Mulsant & Rey and *Gyronycha* Casey (both Aleocharinae: Oxypodini: Meoticina), are recorded from New Brunswick and Canada for the first time. The following species are newly recorded or described as new in New Brunswick and Canada: *Apimela fusciceps* (Casey); *A. canadensis* Klimaszewski & Webster, **sp. n**.; and *Gyronycha pseudoobscura* Klimaszewski & Webster, **sp. n.** The genera are defined and the key for species identification is provided. Color habitus images and black and white images of the median lobe of the aedeagus, spermatheca, tergite, and sternite VIII are provided for all species occurring in Canada, and *Apimela macella* (Erichson), the type species of genus *Apimela*, and *G. valens* Casey, the type species of *Gyronycha*. New or additional habitat data are provided for the species treated in this contribution. The following new synonym is established: *Gyronycha lepida* Casey, 1911 (NC), is a synonym of *G. fusciceps* Casey, 1894 (NC).

## Introduction

The genus *Gyronycha* was described by Casey (1894) to accommodate his seven new species distributed in the USA (CA, NC, NJ, NY, NV, TX). Later, Casey (1911) added two *Gyronycha* species from North Carolina and New York. Casey (1885) described *Calodera attenuata* from California, which was transferred by Seevers (1978) to *Apimela* Mulsant & Rey. The species of *Gyronycha* described by Casey represent a mixed group and most of them belong to the genus *Apimela*, for details see the checklist of *Apimela* and *Gyronycha* further in the text. Moore and Legner (1975) reported three valid Nearctic species of *Apimela*: *A. attenuata* (Casey, 1885) [with *G. lineata* Casey, 1894, as its synonym], *A. fenyesi* (Bernhauer, 1906), and *A. longipennis* (Casey, 1911), all from California. Seevers (1978) redefined the two genera and distinguished *Apimela* from *Gyronycha* by smaller and more slender body, transverse pronotum [both genera have distinctly or slightly transverse pronotum], transverse antennomeres IV-X, presence of a tubercle arising from the margin of first and fifth visible male tergites, and the distinctive form of the spermatheca (Fig. 25E, spermatheca of *A. attenuata* (Casey) in Seevers 1978). Seevers (1978) designated *G. valens* Casey as a type species of *Gyronycha*, because Casey (1894) did not designate specifically one species as the types of the genus. Seevers (1978) synonymized genus *Gyronychina* Casey, 1911, described from one California species (*G. longipennis* Casey), with the genus *Apimela*. The generic type of *Apimela* is the Palaearctic species *A. macella* (Erichson, 1839) [Figs 27–29; male median lobe of aedeagus in lateral view, illustrated by Seevers 1978, Fig. 5f, g]. We have modified Seevers’s (1978) diagnosis in separating *Apimela* from *Gyronycha* when making generic assignment of the new species discovered in New Brunswick (NB). Our modified diagnoses of the two genera are included in the key to species. For all Nearctic species of *Apimela* and *Gyronycha* see below the checklist in this paper.

## Materials and methods

All specimens in this study were dissected to examine the genital structures. Extracted genital structures were dehydrated in absolute alcohol, mounted in Canada balsam on celluloid micro-slides, and pinned with the specimen from which they originated. Images of the entire body and the genital structures were taken using an image processing system (Nikon SMZ 1500 stereoscopic microscope; Nikon Digital Camera DXM 1200F, and Adobe Photoshop software).

Morphological terminology mainly follows that used by Seevers (1978). The ventral side of the median lobe of the aedeagus is considered to be the side of the bulbus containing the foramen mediale, the entrance of the ductus ejaculatorius, and the adjacent ventral side of the tubus of the median lobe with the internal sac and its structures (this part is referred to as the parameral side in some recent publications); the opposite side is referred to as the dorsal part. In the species descriptions, microsculpture refers to the surface of the upper forebody (head, pronotum and elytra).

Species within genera are arranged alphabetically in the text and in the table.

### Depository/institutional abbreviations

**CNC** Canadian National Collection of Insects, Arachnids, and Nematodes, Agriculture and Agri-Food Canada, Ottawa, Ontario, Canada.

**FMNH** Field Museum of Natural History, Chicago, USA.

**LFC** Natural Resources Canada, Canadian Forest Service, Laurentian Forestry Centre, R. Martineau Insectarium, Quebec City, Quebec, Canada.

**RWC** R. Webster collection, Fredercton, New Brunswick, Canada.

**USNM** United States National Museum, Washington, D.C, USA.

USA state abbreviations follow those of the US Postal Service.

**Discussion.** We have discovered that the spermathecal capsule in *Apimela* and *Gyronycha* has an apical or apico-lateral, narrow, tubular projection, which may be indicative of close phylogenetic relationship between both genera. Known males of *Gyronycha* species, have carniform tubercules on the first and fifth visible tergites (Figs 16, 21, 22), and females are lacking these structures. These tubercles are absent in *Apimela*. Seevers (1978) pointed out that *Apimela* and *Meotica* Mulsant & Rey are similar in having small, slender and compressed body but considered *Apimela* to be closely related to *Gyronycha* due to elongate elytra and mesoventrite, and the distinctive form of spermatheca. Externally, species of *Apimela* are very similar to those of *Alisalia* Casey, which live in very similar habitats, but the latter have shorter elytra and mesoventrite and have different type of genitalia (Klimaszewski et al. 2009). Casey (1894) considered *Gyronycha* as allied to Central American *Bamona* Sharp but his hypothesis needs further studies to be confirmed.

### Key to Canadian species of *Apimela* and *Gyronycha*

**Table:** 

1	Antennomeres VII-X slightly to strongly transverse (Figs 1, 9, 27); tarsal claws small (Figs 1, 9); males without tubercles on first and fifth visible tergites; spermatheca with sinuate stem, coils partial and not overlapping (Figs 8, 15, 29) (*Apimela*)	**2**
–	Antennomeres VII-X moderately to strongly elongate (Figs 16, 23); tarsal claws large (Fig. 16); males with strong tubercles on first and fifth visible tergites (Figs 21, 22); spermatheca with broadly and irregularly coiled stem, coils overlapping (Fig. 26) (*Gyronycha*)	***Gyronycha pseudoobscura* Klimaszewski & Webster, sp. n.** [male unknown]
2	Antennomeres VI-X strongly transverse (Fig. 9); elytra slightly broader than maximum width of pronotum (Fig. 9); eyes moderately large (Fig. 9); spermatheca with spherical apical part of capsule bearing elongate and multiply micro-coiled projection (Fig. 15)	***Apimela canadensis* Klimaszewski & Webster, sp. n.**
–	Antennomeres VI-X slightly transverse (Fig. 1); elytra distinctly broader than maximum width of pronotum (Fig. 1); eyes large (Fig. 1); spermatheca with tubular apical part of capsule and with short and few microcoiled projection *Gyronycha* (Fig. 8)	***Apimela fusciceps* (Casey)**

### Taxonomy

#### Apimela

Taxon classificationAnimaliaColeopteraStaphylinidae

Mulsant & Rey, 1874

Figs 1–8 , 9–15 , 27–29

##### Type species.

*Homalota macella* Erichson, 1839

##### Diagnosis.

Body yellowish brown, narrow and linear, length 2.0-3.0 mm; forebody densely and finely pubescent; head subquadrate as large as or slightly larger than pronotum, eyes moderately large, usually shorter than postocular area of head and visible from above, posterior angles of head angular, basal carina vestigial and visible only basally; antennomeres V-X slightly to strongly transverse; last palpomere needle-shaped; pronotum slightly transverse, widest in apical third, as long as head, densely pubescent, pubescence on midline of disc directed anteriad except posteriad basally, on sides anteriad and laterad, forming arcuate lines; elytra strongly elongate, one six/seventh broader than pronotum, at suture longer than pronotum, pubescence directed obliquely postriad; mesoventrite long, mesocoxae close; abdomen parallel sided, first four visible tergites with deep arcuate impressions, males without tubercles on first and fifth visible tergites; basal metatarsus as long as two following combined, tarsi small; median lobe of aedeagus with sinuate venter of tubus in lateral view, crista apicalis of bulbus from moderately-sized to large, internal sac with complex sclerites; spermatheca with sinuate stem, coils partial and not overlapping. Species of this genus occur in riparian habitats.

#### Apimela fusciceps

Taxon classificationAnimaliaColeopteraStaphylinidae

(Casey) comb. n.

Figs 1–8

Gyronycha fusciceps Casey, 1894: 376. **Lectotype** (female). USA: N.Y. [New York];fusciceps Casey; TYPE USNM 38789; Casey bequest 1925; our lectotype designation label (USNM). There is an unpublished Gusarov’s lectotype designation label. The genitalia were probably treated by KOH as they are barely recognizable and the spermatheca is missing. Lectotype - present designation. PARALECTOTYPE: USA: N.Y. [New York]; fusciceps Casey; TYPE USNM 38789, fusciceps-2; Casey bequest 1925; our paralectotype designation label (USNM) 1 male. There is an unpublished Gusarov’s paralectotype label. The genitalia were probably treated by KOH as they are barely recognizable.
Gyronycha lepida Casey, 1911: 217, syn. n. **Lectotype** (male). USA: N.C. [North Carolina]; lepida Casey; TYPE USNM 38790; Casey bequest 1925; our lectotype designation label (USNM). There is an unpublished Gusarov’s lectotype designation label. Lectotype - present designation. PARALECTOTYPES: N.C. [North Carolina]; lepida Casey; TYPE USNM 38790, lepida-2; Casey bequest 1925; our paralectotype designation label (USNM) 1 female [there is an unpublished Gusarov’s paralectotype designation label]; N.C. [North Carolina]; TYPE USNM 38790, lepida-3 Casey; Casey bequest 1925; our paralectotype designation label (USNM)1 female [there is an unpublished Gusarov’s paralectotype designation label].

##### Material examined.

**** Canada, New Brunswick, Carleton Co., Belleville, Meduxnekeag Valley Nature Preserve, 46.1944°N, 67.6832°W, 2.VI.2008, R.P. Webster, coll.// River margin, under cobblestones in sand/gravel, among scattered grasses (RWC, LFC) 2 females; Belleville, Meduxnekeag Valley Nature Preserve, 46.1942°N, 67.6832°W, 9.VI.2008, R.P. Webster, coll. // River margin, under cobblestones among grasses away from water’s edge (RWC) 2 females; Belleville, Meduxnekeag Valley Nature Preserve, 46.1921°N, 67.6815°W, 11.VI.2010, R.P. Webster, coll. // River margin, under cobblestones set in sand (RWC) 1 female; Belleville, Meduxnekeag Valley Nature Preserve, 46.1941°N, 67.6830°W, 31.V.2013, R.P. Webster, coll. // River margin, under small rock (RWC) 1 male.

**Figures 1–8.**
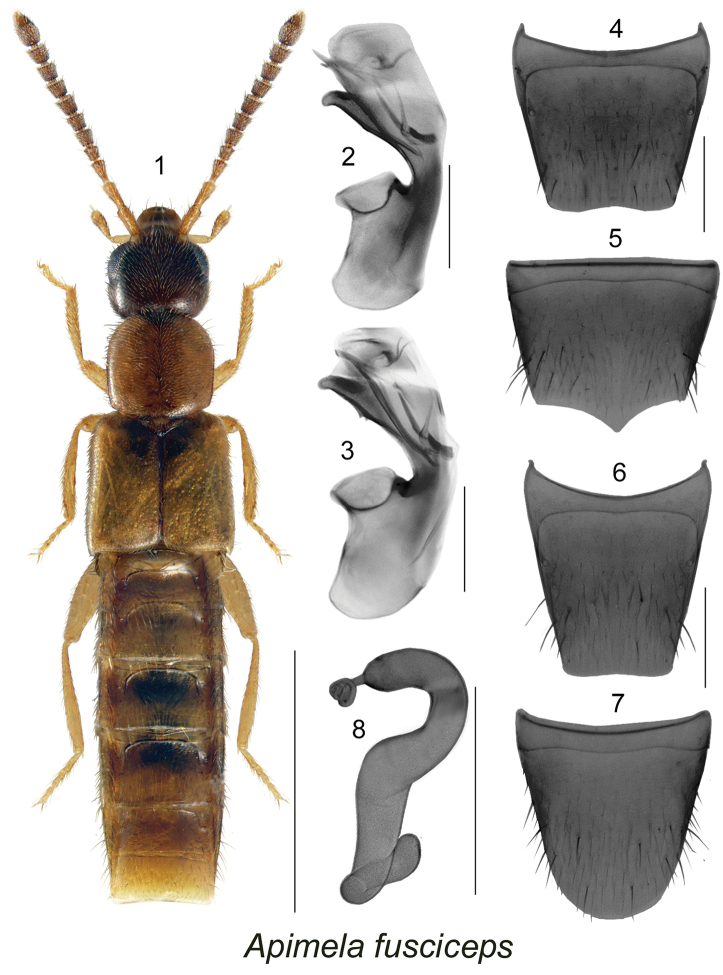
*Apimela fusciceps* (Casey): **1** habitus in dorsal view **2–3** median lobe of aedeagus in lateral view **4** male tergite VIII **5** male sternite VIII **8** spermatheca **6** female tergite VIII **7** female sternite VIII. Scale bars: 1 mm for habitus; 0.2 mm for remaining structures.

##### Diagnosis.

Body length 3.0–3.4 mm, subparallel, yellowish brown, head and scutellar region of elytra dark brown, strongly glossy, forebody with fine and moderately dense pubescence, punctation fine; head subquadrate, eyes large and about as long as postocular region of head, posterior angles rounded, pubescence directed straight and obliquely anteriad; antennomeres V-X slightly to strongly transverse, head broader than pronotum; pronotum slightly transverse, posterior angles angular; elytra elongate, at suture longer than pronotum, and about one fourth wider than pronotum, abdomen subparallel, with first visible four tergites deeply impressed basally, males lacking tubercles on first and fifth visible tergites. MALE. Median lobe of aedeagus with tubus strongly produced ventrally, in lateral view its venter sinuate with two more or less visible minute teeth in apical third, internal sac with complex structures as illustrated (Figs 2 [NB], 3 [holotype]); tergite VIII truncate apically (Fig. 4); sternite VIII produced apically and sharply pointed (Fig. 5). FEMALE. Spermatheca S-shaped, capsule tubular, slightly arched and with apical narrow, tubular projection coiled apically, stem sinuate and twisted (Fig. 8); tergite VIII truncate apically (Fig. 6); sternite VIII rounded apically (Fig. 7).

##### Distribution.

Formerly known from New York and North Carolina in the United States (Casey 1894, 1911). Here, reported in New Brunswick, Canada, for the first time.

##### Collection and habitat data.

In New Brunswick, this species was found along a river margin under cobblestones set in sand/gravel, often in areas with scattered grasses, sometimes away from water’s edge. Adults were collected in late May and June.

##### Comments.

This species belongs to a distinct species group and has spermatheca type similar to that of *A. attenuata* (Casey).

#### Apimela canadensis

Taxon classificationAnimaliaColeopteraStaphylinidae

Klimaszewski & Webster sp. n.

http://zoobank.org/2CAD6FD2-6B89-45A8-93E1-4DE94B359B60

Figs 9–15

##### Holotype

**(male).** CANADA, New Brunswick, Restigouche Co., Jacquet River Gorge PNA, 47.8257°N, 66.0779°W, 14.V.2010, R.P. Webster, coll. // Partially shaded cobblestone bar near outflow of brook at Jacquet River, under cobblestones and gravel on sand (LFC). PARATYPES: New Brunswick, Restigouche Co., Jacquet River Gorge PNA, 47.8257°N, 66.0779°W, 14.V.2010, R.P. Webster coll. // Partially shaded cobblestone bar near outflow of brook at Jacquet River, under cobblestones & gravel on sand (RWC) 2 males, 1 female; Carleton Co., Belleville, Meduxnekeag Valley Nature Preserve, 46.1942°N, 67.6832°W, 9.VI.2008, R.P. Webster coll. // River margin, under cobblestones among grasses away from water’s edge (RWC) 1 female.

**Figures 9–15.**
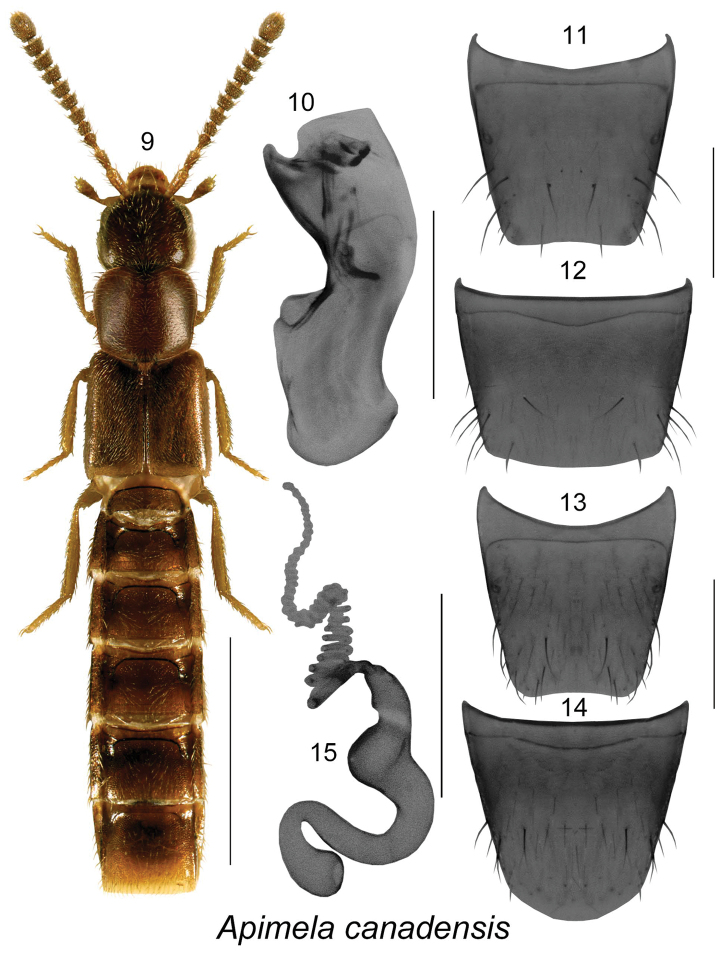
*Apimela canadensis* Klimaszewski & Webster, sp. n.: **9** habitus in dorsal view **10** median lobe of aedeagus in lateral view; 11, male tergite VIII **12** male sternite VIII **13** female tergite VIII **14** female sternite VIII **15** spermatheca. Scale bars: 1 mm for habitus; 0.2 mm for remaining structures.

##### Etymology.

Named after Canada, the country of origin, and to commemorate the 150^th^ anniversary of Canada.

##### Description.

Body length 2.0–3.0 mm, subparallel, yellowish brown with slightly darker head, moderately glossy, forebody with fine and dense pubescence, punctation fine; head subquadrate, eyes moderately large and shorter than postocular region of head, posterior angles rounded and slightly angular, pubescence directed straight and obliquely anteriad; antennomeres V-X strongly transverse, head slightly broader than pronotum; pronotum slightly transverse, posterior angles angular; elytra elongate, at suture longer than pronotum, and about one sixth wider than pronotum, abdomen subparallel, with first four visible tergites deeply impressed basally, males lacking tubercles on first and fifth visible tergites. MALE. Median lobe of aedeagus with tubus strongly produced ventrally, its venter sinuate with apex turned slightly upward, internal sac with complex structures as illustrated (Fig. 10); tergite VIII truncate apically (Fig. 11); sternite VIII truncate and broadly arcuate apically (Fig. 12). FEMALE. Spermatheca S-shaped, capsule spherical, slightly arched with a narrow apical tubular multiple micro-coiled projection, stem sinuate, S-shaped (Fig. 15); tergite VIII truncate apically (Fig. 13); sternite VIII rounded apically (Fig. 14).

##### Distribution.

Known only from New Brunswick, Canada.

##### Collection and habitat data.

The holotype and three paratypes were captured on a partially shaded cobblestone bar near the outflow of brook along the Jacquet River. The adults were found under cobblestones and gravel in sand. One paratype was found along a river margin under a cobblestone among grasses away from the water’s edge. Adults were collected in May and June.

##### Comments.

This species clearly belongs to a different species group than *A. fusciceps*, which has capsule of spermatheca entirely tubular.

#### Gyronycha

Taxon classificationAnimaliaColeopteraStaphylinidae

Casey, 1894

Figs 16–22 , 23–26

##### Type species.

*Gyronycha valens* Casey, 1894.

##### Diagnosis.

Body yellowish brown, narrow and linear, length 2.5–4.2 mm; forebody densely and finely pubescent; head round, as large as pronotum, eyes large, about as long as postocular area of head visible from above, posterior angles of head rounded, basal carina vestigial and visible only basally; antennomeres V-X slightly to strongly elongate; last palpomere needle-shaped; pronotum slightly transverse, widest in apical third, as long as head, densely pubescent, pubescence on midline of disc directed anteriad except posteriad basally, on sides anteriad and laterad, forming arcuate lines; elytra strongly elongate, one fifth broader than pronotum, at suture longer than pronotum, pubescence directed obliquely postriad; mesoventrite long, mesocoxae close; abdomen parallelsided, first four visible tergites with deep arcuate impressions, males with tubercles on first and fifth visible tergites; basal metatarsus as long as the following two combined, tarsi large; median lobe of aedeagus with strongly sinuate venter of tubus in lateral view, crista apicalis of bulbus moderately large, internal sac with complex sclerites; spermatheca with broadly and irregularly coiled stem, coils overlapping. New Brunswick specimens of this genus were found in gravel in a riparian habitat.

**Figures 16–22.**
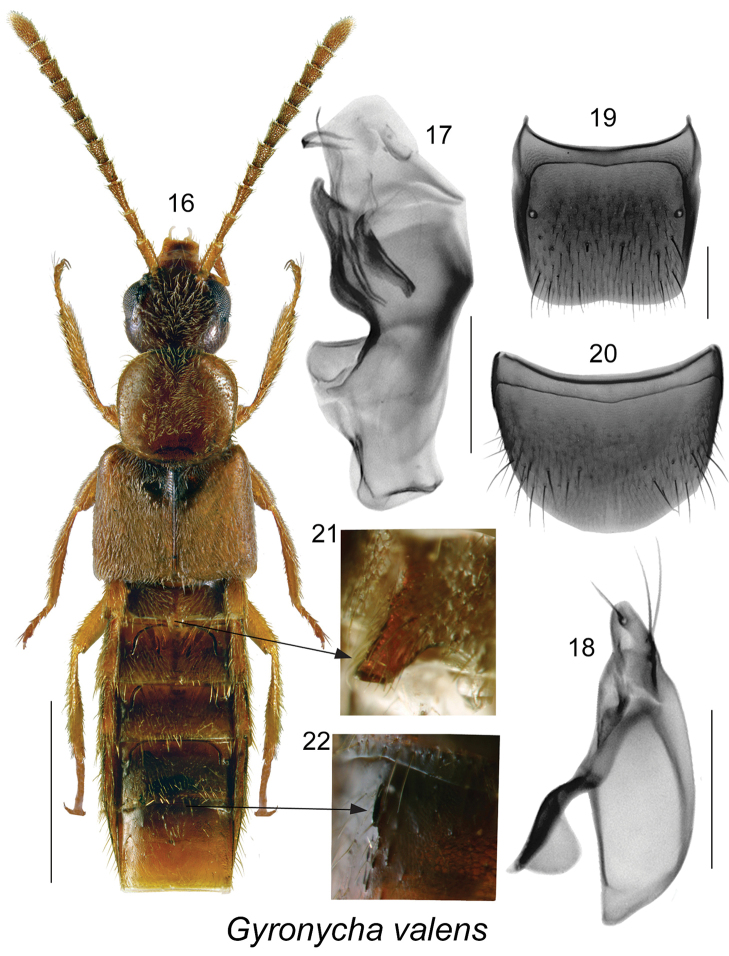
*Gyronycha valens* Casey (type species of the genus): **16** habitus in dorsal view **17** median lobe of aedeagus in lateral view **18** Paramere **19** male tergite VIII **20** male sternite VIII **21** dorsal projection on first visible male tergite; and **22** on fourth visible male tergite. Scale bars: 1 mm for habitus; 0.2 mm for remaining structures.

#### Gyronycha pseudoobscura

Taxon classificationAnimaliaColeopteraStaphylinidae

Klimaszewski & Webster sp. n.

http://zoobank.org/484B63FF-BE0A-4BBF-A0E1-8E71F0D71C49

Figs 23–26

##### Holotype

**(female).** CANADA, New Brunswick, Restigouche Co., Jacquet River Gorge PNA, 47.8257°N, 66.0779°W, 24.V.2010, R.P. Webster coll. // partially shaded cobblestone bar near outflow of brook at Jacquet River, under cobblestones and gravel on sand (LFC). PARATYPE: New Brunswick, Queens Co., Bayard at Nerepis River, 45.4426°N, 66.3280°W, 24.V.2008, R.P. Webster coll. // River margin, in gravel (RWC) 1 female.

**Figures 23–26.**
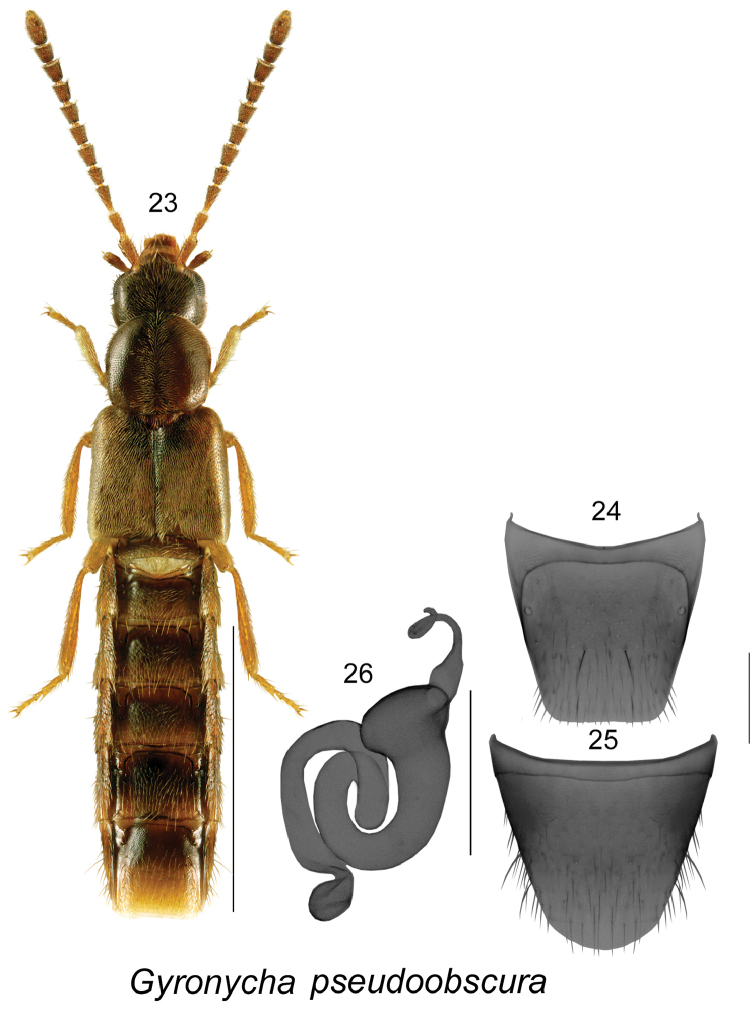
*Gyronycha pseudoobscura* Klimaszewski & Webster (female): **23** habitus in dorsal view **24** tergite VIII **25** sternite VIII **26** spermatheca. Scale bars: 1 mm for habitus; 0.2 mm for remaining structures.

##### Etymology.

The name of this new species, *pseudoobscura*, derives from a similar species of *Gyronycha obscura* Casey described from California, USA.

##### Description.

Body length 3.9–4.0 mm, subparallel, yellowish brown with head and pronotum and antennae dark brown, moderately glossy, forebody with fine and dense pubescence, punctation fine; head round, eyes moderately large and shorter than postocular region of head, posterior angles rounded, pubescence directed straight and obliquely anteriad; antennomeres V-X slightly elongate, head about as broad as pronotum; pronotum slightly transverse, posterior angles slightly angular; elytra elongate, at suture longer than pronotum, and about one fifth wider than pronotum, abdomen subparallel, with first visible four tergites deeply impressed basally. MALE. Unknown. FEMALE. Spermatheca with subspherical capsule, and with apical narrow, tubular and coiled apically projection, stem sinuate, with large, overlapping coils (Fig. 26); tergite VIII truncate apically (Fig. 24); sternite VIII rounded apically (Fig. 25).

##### Collection and habitat data.

The holotype was captured under cobblestones and gravel on sand on a partially shaded cobblestone bar near the outflow of a brook flowing into the Jacquet River. The paratype was captured in gravel along a river margin.

##### Comments.

This species is similar externally and has similar shape of spermatheca and female tergite and sternite VIII to those of *G. obscura* Casey. *Gyronycha pseudoobscura* may be distinguished from *G. obscura* by narrower body, dark brown color of head and pronotum (light brown in *G. obscura*), and the differently shaped pronotum with anterior angles rounded and strongly converging apically in apical part of the disc, while the pronotal angles are rectangular and moderately converging apically in *G. obscura*. The two species have allopatric distribution, and are known from remote and disjunctive localities in New Brunswick, Canada, and California, United States of America.

**Figures 27–29.**
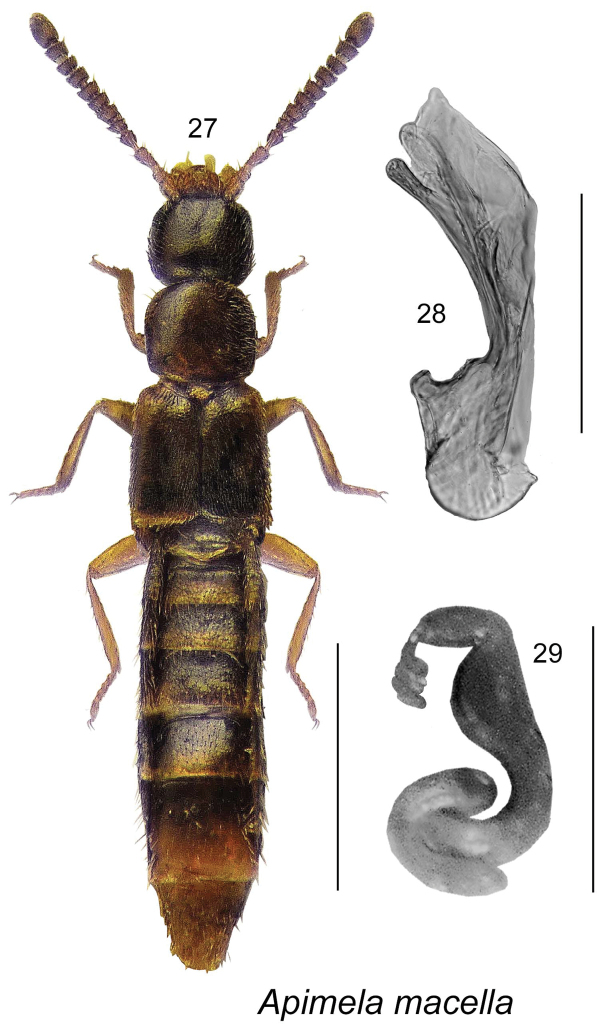
*Apimela macella* (Erichson) (type species of the genus from Europe): **27** habitus in dorsal view **28** median lobe of aedeagus in lateral view **29** spermatheca. Scale bars: 1 mm for habitus; 0.2 mm for remaining structures.

### Checklist of *Apimela* and *Gyronycha* species in Canada and USA, valid species and new records are in bold

**Table:** 

**Taxon**	**Author**	**Original generic assignment**	**Distribution**	**Present taxonomic status**
***Apimela attenuata***	Casey, 1885: 306	*Calodera*	CA, NV	Valid species [spermatheca illustrated by Seevers 1978: 207]
***Apimela lineata***	Casey, 1894: 376	*Gyronycha*	NV	Synonymized with *A. attenuata* by Moore and Legner (1975): 343. As valid in Seevers 1978: 252).
***Apimela fenyesi***	Bernhauer, 1906: 337	*Aleuonota*	CA	Valid species: Moore and Legner 1975: 343; confirmed here. As valid in Seevers (1978: 252).
***Apimela fusciceps***	Casey, 1894: 376	*Gyronycha*	**NB**, NC, NY	Valid species. **New combination**
***Apimela lepida***	Casey, 1911: 217	*Gyronycha*	NC	**New synonymy**
***Apimela longipennis***	Casey, 1911: 219	*Gyronychina*	CA	As valid species Moore & Legner 1975: 343, Seevers (1978: 252).
***Apimela longicornis***	Casey, 1911: 217	*Gyronycha*	NC, NY	Tentatively affiliated with *Apimela*. **New combination**
***Apimela pertenuis***	Casey, 1894: 377	*Gyronycha*	NJ	Status uncertain, not examined. **New combination**
***Apimela canadensis***	Klimaszewski & Webster, sp. n.	*Apimela*	**NB**	Valid species
***Gyronycha obscura***	Casey, 1894: 375	*Gyronycha*	CA	Male unknown, species tentatively affiliated with *Gyronycha*
***Gyronycha pseudoobscura***	Klimaszewski & Webster, sp. n.	*Gyronycha*	**NB**	Male unknown, species tentatively affiliated with *Gyronycha*
***Gyronycha texana***	Casey, 1894: 374	*Gyronycha*	TX	Valid species
***Gyronycha valens***	Casey, 1894: 373	*Gyronycha*	AZ, CA, IN, NC, NM, NY, SO, TX	Valid species

## Supplementary Material

XML Treatment for Apimela

XML Treatment for Apimela fusciceps

XML Treatment for Apimela canadensis

XML Treatment for Gyronycha

XML Treatment for Gyronycha pseudoobscura
